# Recent Advances in Functional Polymer Materials for Energy, Water, and Biomedical Applications: A Review

**DOI:** 10.3390/polym13244327

**Published:** 2021-12-10

**Authors:** Yassine EL-Ghoul, Fahad M. Alminderej, Fehaid M. Alsubaie, Radwan Alrasheed, Norah H. Almousa

**Affiliations:** 1 Department of Chemistry, College of Science, Qassim University, King Abdulaziz Rd, P.O. Box 1162, Buraidah 51452, Saudi Arabia; 2 Textile Engineering Laboratory, University of Monastir, Monastir 5019, Tunisia; 3 National Center for Chemical Catalysis Technology, King Abdulaziz City for Science and Technology, P.O. Box 6086, Riyadh 11442, Saudi Arabia; nalmousa@kacst.edu.sa; 4 National Center for Desalination & Water Treatment Technology, King Abdulaziz City for Science and Technology, P.O. Box 6086, Riyadh 11442, Saudi Arabia; ralrasheed@kacst.edu.sa

**Keywords:** polymer/functional polymer applications, batteries, solar cells, water treatment, enhanced oil recovery, biomedical

## Abstract

Academic research regarding polymeric materials has been of great interest. Likewise, polymer industries are considered as the most familiar petrochemical industries. Despite the valuable and continuous advancements in various polymeric material technologies over the last century, many varieties and advances related to the field of polymer science and engineering still promise a great potential for exciting new applications. Research, development, and industrial support have been the key factors behind the great progress in the field of polymer applications. This work provides insight into the recent energy applications of polymers, including energy storage and production. The study of polymeric materials in the field of enhanced oil recovery and water treatment technologies will be presented and evaluated. In addition, in this review, we wish to emphasize the great importance of various functional polymers as effective adsorbents of organic pollutants from industrial wastewater. Furthermore, recent advances in biomedical applications are reviewed and discussed.

## 1. Introduction

An increasing interest in the development of functional materials has led to the appearance of so-called smart polymers, which have demonstrated their practical performance in a wide range of application fields. These technical polymers are successfully gaining a growing number of recipients in the field of renewable energies, medical diagnostics, water treatment, pollution control, environmental protection, and food safety, thanks to their high sensitivity, diversity, specificity, and capacity for analysis in real time [1,2,3,4,5,6,7]. Some polymers are active and functional in nature, but others need to be modified to improve their impact and functionality. Several recent methods and techniques have been developed for the functionalization of the surfaces of synthetic and natural polymers [8,9,10,11,12]. Indeed, the terminal groups of the surface of a polymer could be linked or modified by reactive functional groups. Secondly, different molecules, oligomers, or active/bioactive polymers can be grafted to the surface, thus offering new desired properties which match the requirements of a targeted use [13,14,15,16,17]. Because of their inert character, polymeric surfaces need to be pre-activated before proceeding to their functionalization. This pre-treatment will give them an active surface for the immobilization of the various active agents. This surface activation could be performed chemically by grafting different active functions and branches, or physically, via different techniques, such as plasma treatment, laser treatment, UV irradiation, ozonolysis, electron beams, etc. [18,19,20,21,22,23,24]. Functionalization of polymeric surfaces is generally provided chemically either via covalent bonds and low energy interactions [25,26,27], or by non-covalent physical attraction, such as the adsorption of pollutants [28,29], antibacterial biomaterials [30,31,32], and drug delivery systems [33,34,35]. Covalent chemical functionalization remains the most interesting and the most advantageous. Indeed, it ensures a good durability of the active ingredients and a good stability of the active principle before and after its applied action. The chemical grafting of polyfunctional molecules or macromolecules and the functionalization via spacer compounds increases the efficiency of the polymeric surfaces by conferring them more active and spaced functions, therefore making them more effective and relevant.

**Figure 1:**
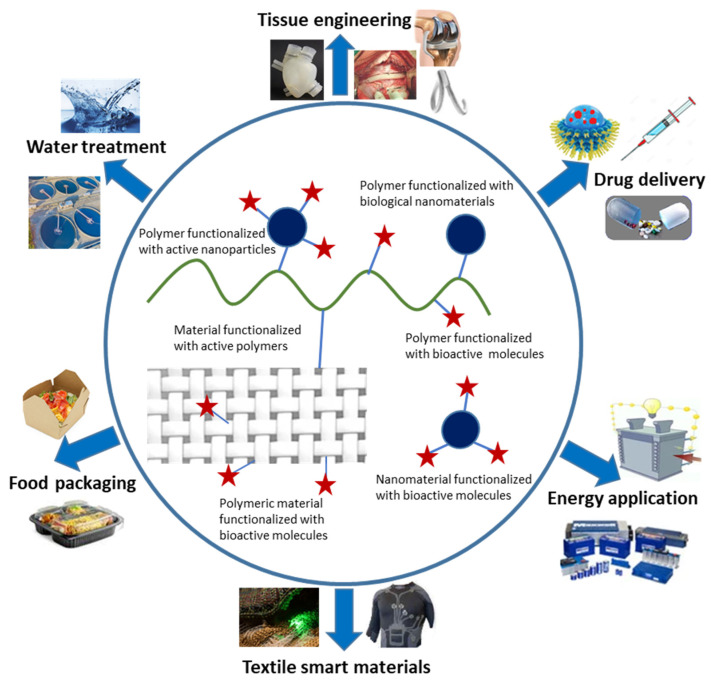
Applicative fields related to functional polymeric materials.

Below is an overview of recent advances in polymers and functional polymeric materials and their exploration in the development of various applicative fields and industrial equipment (Figure 1).

## 2. Energy Applications of Polymers

**Figure 2:**
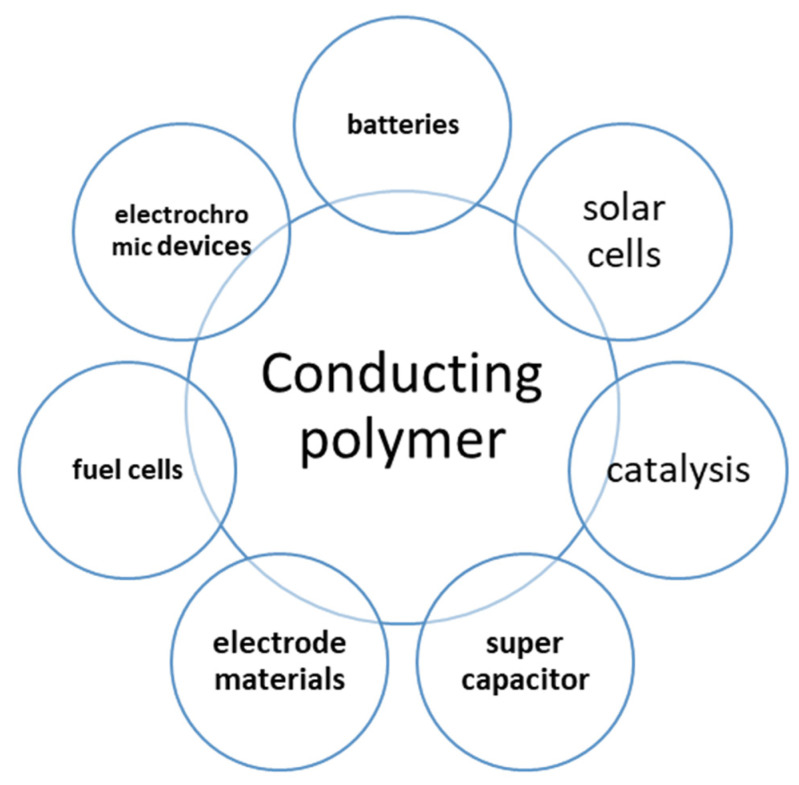
General applications of conducting polymers.

Currently, energy and sustainable energy have increasingly gained a leading position as the most important global concerns in view of the increased depletion of fossil fuels. Material and nanomaterial-based polymers and their composites are investigated in many various applications related to energy storage and production (Figure 2), including batteries, solar cells, super-capacitors, domestic tools, vehicles, fuel cells, biomedical equipment, and surgical appliances [36,37,38,39,40,41,42,43,44]. Conducting polymers are organic polymers that can conduct electricity, and they also may be used as semiconductors. Generally, the class of polymers known as characteristically conducting polymers, or electroactive conjugated polymers, were developed about 20 years ago, and their ability to conduct electricity is due to the occurrence of delocalized molecular orbitals. In addition to their conduction properties, they also exhibit interesting characteristics, such as electronic, magnetic, wetting, optical, mechanical, and microwave absorption properties. Conducting polymers (CPs) have received a lot of attention due to their economic importance, good environmental stability, and electrical conductivity, as well as their useful mechanical, optical, and electronic properties. Generally, conducting polymers have different nanostructures with a higher specific capacitance and may constitute an alternative in the development of new-generation energy storage devices [45,46,47,48,49,50]. There are many types of conducting polymers that have the ability to conduct electrical current. These conducting polymers generally are classified into three principal groups: ionic conducting polymers [51,52,53], intrinsically conducting polymers (ICPs), which also are known as synthetic metals [54,55,56] and conducting polymer composites [57,58,59,60].

This distinctive type of polymer has been used in many important applications in the fields of the production and storage of energy, such as in energy assembly, energy storage, solar cells, batteries, photocatalysis materials, electrode materials, electrochromic devices, dye-sensitized electric cells, light emitting and sensing devices, and perovskite electric cells. They also have been used in other important applications, including as p-type conducting parts in thermoelectric generators, as well as being the polymer composites that are used in thermoelectric generators, piezoelectric materials, triboelectric generators, and super capacitors [61,62,63,64,65,66,67,68,69,70,71,72,73,74]. Figure 2 shows the general applications of these conducting polymers.

Polyacetylene, polypyrrole, polythiophene, and polyaniline are examples of intrinsically conducting polymer ICPs. Among the existing conducting polymers, polyaniline has attracted considerably more attention than other types of polymers in recent years because of its superior properties, which include its ease of synthesis, unvarying conduction mechanism, and superior resistance to the effects of oxygen and water [75,76,77].

Recently, new types of conducting polymers have appeared and have proven to be effective in several fields and applications.

### 2.1. Batteries as an Energy Storage Application of Polymers

Many organic polymers can retain and store energy when they charged with electric current, and this energy can be used when it is needed, making it a general area for continuous and sustainable investment in both the short term and the long term. Currently, the most common battery systems are based on the Li-ion technology. This technology was proposed by M. S. Whittingham in 1976, and it was commercialized by SONY in 1990. Additionally, in the 1980s, conducting polymers were extolled as promising materials for the next generation of environmentally benign and efficient batteries. In the late 1980s, Bridgestone-Seiko and VARTA/BASF initiated their sales of commercial batteries that were based on polypyrrole and polyaniline, respectively [78]. One of the most intensively studied conjugated polymers for energy storage applications is polypyrrole, which also was used as an anode material to manufacture an aqueous Li-ion battery in conjunction with a LiCoO_2_ cathode [79,80]. Polythiophene has been of interest to electrochemists for decades. The first battery with polythiophene as an active material was produced and described in 1983. Recently, poly(3′-styryl-4,4″-didecyloxyterthiophene), with a maximum capacity of 45 Ah kg^−1^, and poly(4,4″-didecyloxyterthiophene), with a maximum capacity of 95 Ah kg^−1^, were used as anode materials in combination with a polypyrrole cathode. Another type of polymer that was used in an earlier period consisted of polyaniline (PANI) pellet electrodes with different redox states. In addition, polyacetylene usage for anodes and cathodes and a PEO-based electrolyte were presented in 1981. Also, as recently shown by Zhu et al. [81], the bipolar active material known as poly(para-phenylene) can act as both a cathode and an anode. Many organic polymers can retain and store energy when charged with electric current, and the energy can be used when needed, making it a general area for continuous and sustainable investment in the short and long term. The ultrafast high energy density, long-term stability, and charge–discharge behavior are unique features of supercapacitors, which have attracted considerable attention recently. Different supercapacitors have emerged as efficient energy storage devices, showing wide applications in several fields, including electric vehicles and continuously automatic production power supplies, etc. [82,83,84,85,86]. These supercapacitors exhibit a higher specific power when compared to lithium-ion batteries. The electrodes of these supercapacitors are materials that are based on metal oxides but mainly on conductive polymers [87,88,89,90]. These conductive polymers have shown an excellent specific capacity and their low cyclic stability has been lately overstated by the investigation of nanocomposites which was based on conducting polymers [91,92,93].

### 2.2. Solar and Fuel Cells as an Energy Production Application of Polymers

Natural resources will be exploited for a clean environment and a good life in different countries. The importance of solar cells in the production of clean and sustainable electric power is attributed to places that government services do not reach or when the production of energy from the sun becomes cheaper than other sources. Silicon solar cells are widely used, but there is considerable research being done with the aim of providing less expensive solar cells, such as polymer solar cells and perovskite solar cells. [94,95,96,97].

Polymer solar cells, also known as plastic solar cells, use conjugated polymers as light absorbers, electron donors, electron acceptors, and/or hole transport materials, and these solar cells have been investigated for twenty years. A typical polymer solar cell contains a donor/acceptor bulk-heterojunction, a light-harvesting layer that is sandwiched between the electron and hole extraction layer, then the anode and the cathode. When polymer solar cells were first developed, their structure was similar to a conventional silicon-based solar cell with a planar junction. People believe that this device works as a P-N junction solar cell, based simply on its organic p-type and n-type semiconductor material coatings. At this point, the polymer functions as a photoactive layer for light absorption, charge generation, and transport [98,99].

Nowadays, various electrochemical reactions have been investigated in the direct conversion of chemical energy into electricity, in the context of fuel cells. These fuel cells have recently experienced great progress in their application for the production of electric vehicles [100,101]. Indeed, direct methanol fuel cells (DMFCs) have shown great potential in various energy applications, due to their energy conversion performance, high fuel portability, and eco-friendly aspects [102,103,104]. Several parameters influencing the efficiency of DMFCs have been reported, and the effects of the electrocatalysts used have been widely studied. These electrocatalysts are mainly conducting polymers, having primarily 1D and 2D nanostructures [105,106,107,108,109,110].

## 3. Oil and Gas Applications

Enhanced oil recovery (EOR), also known as tertiary recovery, is the most commonly used method to extract crude oil from an oil field when it cannot be extracted otherwise [111,112]. EOR can extract 30 to 60% or more of the oil from a reservoir. Due to the decrease in the discoveries of oil over the past few years, it is believed that enhanced oil recovery technologies will be vitally important, by ensuring the extended use of oil to generate energy. One of the reasons for this is due to the shortage of current oil resources and the difficulty associated with identifying new oil fields. Polymers have an important role in the application of enhanced oil recovery technology, especially surfactant and hydrogel polymers. Surfactant polymers are injected into the reservoir to reduce the interfacial tension between oil and water, which allows recovery of the oil that is trapped by the rocks in the reservoir, thereby increasing the production of oil. A hydrogel polymer is injected into the reservoir to increase the viscosity of the fluid that contains water, making that fluid more difficult to flow than the oil, thereby increasing the production of oil. The most common polymer that is used for this application is one or more of the polyacrylamide group [113,114]. A typical polymer flood project involves the mixing and injecting of polymer over an extended period of time until about 30 to 50% of the pore volume of the reservoir has been injected. The addition of polymer into the reservoir increases the viscosity of water and reduces the relative permeability of the water in the reservoir, thereby increasing the recovery of oil due to the increase in the fractional flow.

Hydrogel polymers have been used for many years to control the mobility of the injected water during enhanced oil recovery applications. These polymers are non-Newtonian (also called pseudoplastic) fluids because their viscosities are a function of the shear rate. They usually are used with surfactants and alkali agents to increase the sweep efficiency of the tertiary recovery floods [115,116,117]. It is important to select the appropriate polymer for a given area. Thus, the permeability of the reservoir and the viscosity of the oil are used to determine which polymer has the optimum molecular weight. The composition of the rock and the extent of adsorption of the polymer are used to determine the best degree of hydrolysis.

### 3.1. Polyacrylamides

The synthetic polymer used in enhanced oil recovery applications is almost always one of the polyacrylamides. A variety of these products is available from several manufacturers. In general, the performance of a polyacrylamide depends on its molecular weight and its degree of hydrolysis [113,114,118]. Partially hydrolyzed polyacrylamide (HPAM) is one of the polyacrylamide group, and it has the shape of a straight chain polymer of acrylamide monomers, some of which have been hydrolyzed. The HPAM is the polymer most often used in enhanced oil recovery applications, due to its relatively low price and good viscosifying properties [119].

### 3.2. Xanthan Gum/Biopolymer

Xanthan gum is a manufactured polysaccharide that is generally referred to as a biopolymer. It is produced by the microbial action of xanthomonascampestris on a substrate of carbohydrate media, with a protein supplement and an inorganic source of nitrogen. It is well known that xanthan gum has an excellent performance in high salinity brine. It is relatively compatible with most surfactants and the other injection fluid additives which are used in tertiary oil recovery formulations. The biopolymer is usually injected along with an effective biocide, to prevent microbial degradation [120]. Recently, a supramolecular system by self-assembly of xanthan gum with anionic or cationic surfactants and β-cyclodextrin has been developed. This composite polymer system has shown thermal and bio-stability, and greater viscoelasticity in brines, and thus confirmed its potential as a promising tool for enhanced oil recovery applications [121].

### 3.3. Superabsorbent Polymer Composites for Enhanced Oil Recovery

Superabsorbent polymer composites are three-dimensionally crosslinked hydrophilic polymers reinforced by clay, and they are capable of swelling and retaining huge volumes of water in this swollen state [122,123]. Superabsorbent polymer composites have been used as plugging agents in some oil fields in China to meet the need of enhanced oil recovery [124]. After operating for a year, in which water flooding was a perpetual problem, the water content in the crude oil increased, and this decreased the oil output. The high water content in crude oil can cause many problems, such as increased corrosion, increased amounts of sand, and the formation of emulsions that must be disposed of. Based on the results of this research, it was concluded that, when compared to the existing polymer, the superabsorbent polymer composite had good mechanical, thermal, and rheological properties. Recently, pH-sensitive poly (acrylamide-co-methylenebisacrylamide-*co*-acrylic acid) hydrogel microspheres immobilizing silica nanoparticles have been synthesized by reverse suspension polymerization. The prepared hybridized polymeric composite exhibited a significant improvement in the swelling property as a function of the change in pH and showed a 23% increase in the oil recovery factor [125]. Even so, additional advanced studies should be done to determine whether these different polymers could be used effectively for enhanced oil recovery [126].

## 4. Advances in Biomedical Applications

**Figure 3:**
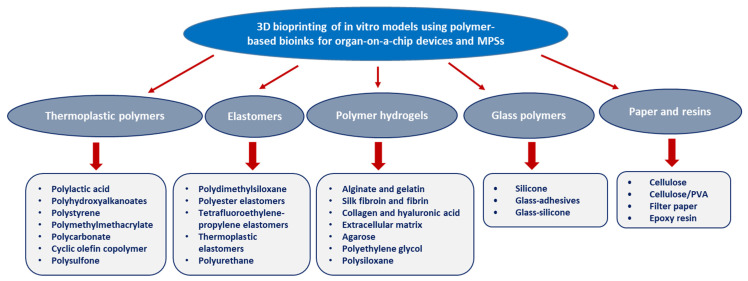
Polymer-based bioinks used for organ-on-a-chip platforms and microphysiological systems (MPSs).

The biomedical sector is a very specific field of applications for polymeric materials. Indeed, it exploits, or attempts to exploit, various compounds by controlling their different chemical, physical, and mechanical properties while ensuring an effective therapeutic function towards complex biological systems and phenomena, whose parameterization is almost impossible. For a long time, man has tried to exploit the macromolecular compounds he has invented and developed over the years for various therapeutic purposes. Thus, since the Second World War, the biomedical field, comprising a surgical component involving prosthetic systems, and a pharmacological component, implying drug substances, was a booming scientific and economic sector. Polymers and functionalized polymeric materials are widely studied in many biomedical applications mainly due to their relevant properties, excellent biocompatibility, and the diversity of their technical characteristics. Many polymers and functional polymeric materials have been developed to improve the performance of medical diagnostics through various approaches, including the enhancement of the contrast in imaging technologies and the promotion of molecular recognition in advanced diagnostic assessments. Polymers for diagnostics have attracted the attention of researchers and manufacturers considerably, due to their reliability in offering both simple and rapid diagnostics, as well as in the transport and protection of drugs immobilized in their structures. Vallejos et al. have prepared a polymeric chemosensory membrane-based vinyl copolymer, grafted with 6-methoxyquinoline groups as chloride responsive fluorescent moieties. This sensory material revealed an excellent efficacy for the detection and quantification of chloride in human sweat and has thus shown its promising capacity for the diagnosis of cystic fibrosis [127]. Polydimethylsiloxane (PDMS) was the more investigated material in the preparation of different organ-on-a-chip devices and microphysiological systems (MPSs). PDMS has shown an efficient and versatile performance in various medical applications. However, many deficiencies have lowered its importance, and applicative improvements still remain. Recently, various alternative polymeric materials (Figure 3), including hydrogels, elastomers, glass polymers, resins, paper, thermoplastic polymers, and nanocomposites, were applied as organ-on-a-chip devices and microphysiological systems providing more functionalities, such as enhanced inhibition of absorption, leaching, and auto-fluorescence, as well as affording more capacity for rapid prototyping [128,129,130].

These materials were thus promising in personalized medicine, modeling, drug discovery, in vitro pharmacokinetic/pharmacodynamics, and in the investigation of cellular responses to drugs. Indeed, advances in preparation technologies, such as 3D bioprinting, have led to an effective use of different hydrogel-based devices. Various hydrogel-based natural polymers (alginate, gelatine, silk fibroin, hyaluronic acid, collagen, fibrin, decellularized extracellular matrix, and agarose) and synthetic polymers (mainly polyethylene glycol and polysiloxane) have been used as bioinks in the preparation of different materials for building 3D cell-laden structures by bioprinting technologies. Hydrogels can also be used in complex structures and topographies to better mimic the natural extracellular environment. In a recent study, Gebeyehu et al. [131] prepared a polysaccharide hydrogel ink-based 3D bioprinted tumor model for chemotherapeutic drug screening. The fabricated cell-laden scaffolds using the different bioinks (H4 and H4-RGD) exhibited excellent mechanical properties. Bioinks showed a good printability at relatively low temperatures, and without a UV curing step. Xenograft cells (PDC), derived from 3D printed non-small cell lung cancer (NSCLC) patients, exhibited a relatively rapid spheroid growing and the creation of a tumor microenvironment after 7 days. The half maximal inhibitory concentration (IC50) evaluation revealed a greater resistance of 3D spheroids to docetaxel, doxorubicin, and erlotinib to untamed type triple-negative breast cancer (MDA-MB-231-WT) and pulmonary adenocarcinoma (HCC-827) cells. Different results of the shape fidelity, flow property, biocompatibility, and scaffold stability of the H4-RGD hydrogel system confirmed the ability of this natural bioink polymer to be effective in the fabrication of different 3D cell bioprinting models, as well as in the development of in-vitro tumor microenvironments for the high-throughput screening of diverse anticancer drugs. Lin et al. [132] developed 3D bioprinted proximal vascularized tubule models, featuring adjacent ducts lined with a confluent epithelium and endothelium, continuous in a polymeric permeable ECM. Three-dimensional fabricated kidney tissue ensured the timely quantification of glucose reabsorption and albumin uptake over time. The three-dimensional renal tissue, through the different assessments, could provide a valuable platform for various in vitro investigations of renal function, disease modeling and pharmacology.

**Figure 4:**
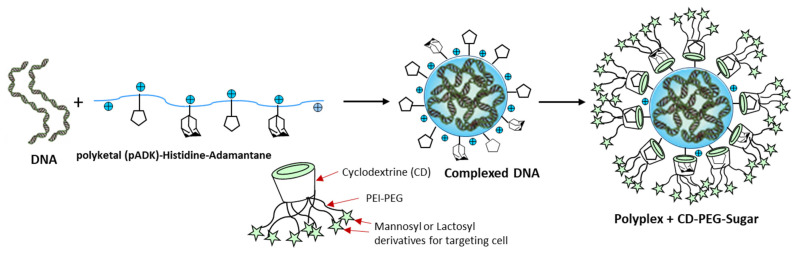
Illustration of smart-shaped polyketal-histidine-adamantane polymer carrier complexing DNA, with a PEG polymer as protective agent and lactose or mannose as cell-targeting molecules, applied in gene therapy.

Currently, functional polymers and are experiencing a rapid and continuous progress as drug delivery and protein purification systems. Due to their diversity, surface and bulk properties, polymers are the most effective biomaterials applied in drug formulations and in drug delivery devices such as implants [133,134,135]. Recent drug delivery systems involve dendrimers, micelles, polymeric nanoparticles, liposomes, microcapsules, cell ghosts, and lipoproteins. These advanced polymeric systems provide a promising tool in the improvement of the intrinsic bioavailability, the safe carrying, the controlled release, and the targeting properties via various mechanisms [136]. Dendrimers are water-soluble, highly structured, designed polymer macromolecules. They are widely investigated as carriers for anti-tumor drugs [137]. PAMAM (polyamidoamine) dendrimers are being studied as efficient carriers (vectors) in gene therapy [138,139,140]. They are synthesized in different sizes and shapes, thus providing well-shaped carriers designed with nanometric sizes. These dendrimers can contain various polymeric ligands, such as PEG, allowing the protection of the ingredients and others, ensuring the targeting of the cells via specific bonds to the cellular receptors, those being essentially via the sugars [141,142]. Linear and branched polyethylenimine (PEI) polymers have shown an excellent performance in DNA encapsulation and complex transfection in gene therapy applications [143]. Adamantane or histidine moieties could be grafted to the polyketal (pADK) polymer to obtain stable DNA complexes and by the inclusion of these moieties in cyclodextrin cavities which are linked to PEI polymer [144]. This modified polymer could be linked to a PEG-sugar-carrier-targeting polymer to finally obtain an efficient star-shaped vector, applied in gene therapy (Figure 4).

A novel gene vector, PEG-GO-PEI-FA-based graphene oxide (GO), was synthesized, in which folic acid (FA) can specifically bind to the folate receptor. Well-condensed and stable nanocomplexes of siRNA were obtained, exhibiting a mild cytotoxicity with a high uptake efficiency in ovarian cancer cells [145].

**Figure 5:**
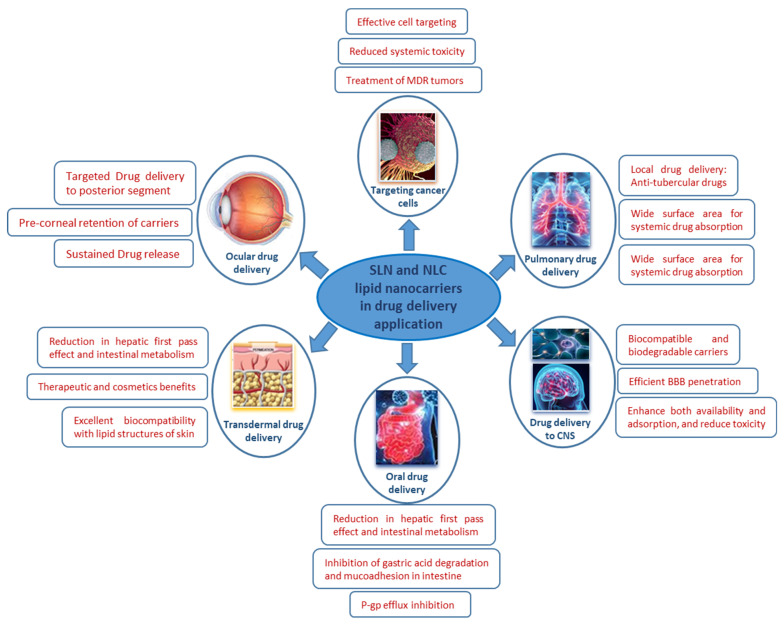
An overview of lipid nanocarriers as drug delivery systems in various therapeutic applications.

Smart polymers display chemical, physical, or structural changes based on changes in the environmental conditions. In biology applications, stimuli-responsive polymers undergo a change in their intrinsic properties in response to the change in biological conditions [146,147,148,149,150,151,152,153,154,155]. The different stimuli can be pH, temperature, pressure, electric or magnetic fields, concentration, light, ionic force, redox potential, etc. [156,157,158,159,160,161,162,163,164,165,166]. During the GI (gastrointestinal) process, the pH changes, which is taken into consideration in the design of different oral drug delivery systems [167,168]. Swollen tissue and cancerous tissue exhibits a significant change in pH. Due to this change in pH, the polymers coating the drugs release their active ingredients. Thanks to this release mechanism, polymers such as the PEI-PEG copolymer release complexed DNA once inside the cell. Similarly, poly (methacrylic acid), linked to the PEG polymer, protects and then releases the proteins that are administered orally. Likewise, polymers which are sensitive to temperature variation, revealing a change in the hydrophilicity/hydrophobicity balance, thus induce a more improved membrane permeation. Among the thermo-responsive polymers, PNIPAAm has been well reported [169,170,171]. The change in temperature causes it to experience an abrupt phase transition. In fact, this polymer has a typical lower critical solution temperature (LCST), below which it exists in the form of a hydrophilic coil; exceeding this temperature, its chain structure suddenly turns into a hydrophobic globule. Recently, a wide range of functional and well-designed polymers were investigated as effective and novel nanocarriers for drug delivery systems in the loading of various natural active metabolites. Besides, lignin nanoparticles (LNPs) were used as nanocarriers of curcumin ingredients, applied as a novel oral drug delivery system. In vitro and in vivo results of their ingredient stability, bioavailability, cell viability, cellular uptake, and intestinal permeation were efficient and promising [172]. By increasing drug solubilization in the stomach and reducing first-pass metabolism via drug diffusion through the lymphatic to the circulatory system, lipids, loading natural ingredients, thereby promote the penetration of these drugs into the digestive tract [173]. Currently, lipid nanoparticles are widely investigated as efficient nanocarriers in different drug delivery applications (Figure 5). Among those lipids used as drug nanocarriers, we find the solid lipid nanoparticle (SLN) which is a colloidal drug nanocarrier, and is generally treated with an emulsifier to improve the stabilization of the prepared dispersion comprising the solid lipid that is melted in water [174]. The SLN, loaded with puerarin, is the most studied complexed nanocarrier. The in vivo assessments on rats revealed its good bioavailability, rapid absorption, and increased tissue concentrations in the heart and brain as targeted organs [175,176]. Zhang et al. developed a new triptolide-loaded SLN nanocarrier system. This organic nanocarrier showed solubility and bioavailability improvements as well as excellent antioxidant and anti-inflammatory effects via the reduction recorded in glutathione (GSH) and myeloperoxidase (MPO) metabolism [177].

Nanostructured lipid carrier (NLC) is a second-generation lipid nano-sized particle, comprising a blend of various solid and liquid lipids [178,179,180,181]. Thymoquinone (from Nigella sativa)-loaded NLC nanocarriers showed an improved bioavailability and oral delivery behavior in 4T1 bearing Balb/C mice. Furthermore, measurements of liver biomarkers and anti-oxidant capacity revealed significant enhancements [182]. Currently, different innovative NLC lipids have been investigated as effective nanocarriers for anticancer purposes. Indeed, citral (from Cymbopogon citratus) [183] and zerumbone (from Zingiber zerumbet L. Smith) [184], as a valuable drug, considerably increased the antitumor activity in lymphoblastic leukemia and breast tumor cells after in vitro and in vivo assessments.

**Figure 6:**
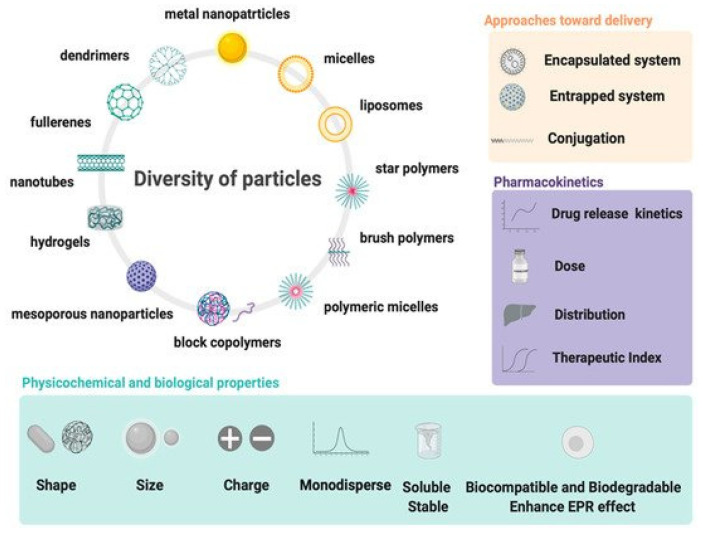
Design and properties requirements for drug delivery systems using wide spectrum of particles. Reprinted with permission from ref. [185]. Copyright 2021 MDPI.

Nanocarrier-based nanoparticles linked to different polymeric ligands were investigated as drug delivery across the blood-brain barrier [186].

Today, oxidative stress is implicated in most brain diseases, and intensifies the impact of tumor tissue. The bioactive polymer mPEG has recently shown its ability to respond to a stimulus of reactive oxygen species (ROS) with a ROS-cleavable moiety (thioketal) connecting the medical ingredient to the polymer [187]. This would increase the ability to target drugs, especially for those sites showing significant oxidative stress. Nanogels that are based on protective polymers have been effective in inhibiting amyloid β (Aβ) fibril formulations [188,189], resulting in a reduced Aβ cell toxicity in vitro [190]. In vivo inoculation of zinc-loaded polymeric poly(lactic-co-glycolic acid) (PLGA) nanoparticles with g7 ligand (g7 is a glycopeptide used for its ability to cross the BBB) for BBB (blood-brain barrier) crossing revealed a significant reduction of plaque size recently [191,192].

The role of functional polymers and diverse forms of particles in drug delivery will expand considerably in the future to treat various unresolved issues (Figure 6). These problems may involve site-specific drug delivery in subcellular organelles, exploiting effectively the chemical, physical, and biological properties with the aim to optimize drug delivery behavior. Nanocomposites have been shown to penetrate deep blood-brain-barriers [193,194]. Nowadays, more knowledge is still needed regarding the biology of cell–polymer interactions, nano-safety, and industrial manufacturing.

Actually, bioactive natural polymers, such as chitosan, alginate, carrageenan, and various polysaccharide extracts from plants were grafted onto various textile biomaterials, including implants and wound dressings. Successful chemical grafting without altering the different mechanical, swelling, and microbiological properties of the grafted biopolymers afforded excellent and promising functionalized textile biomaterials with enhanced physical, bacterial, and biological performance [195,196,197,198,199,200,201,202,203,204,205,206,207,208].

**Figure 7:**
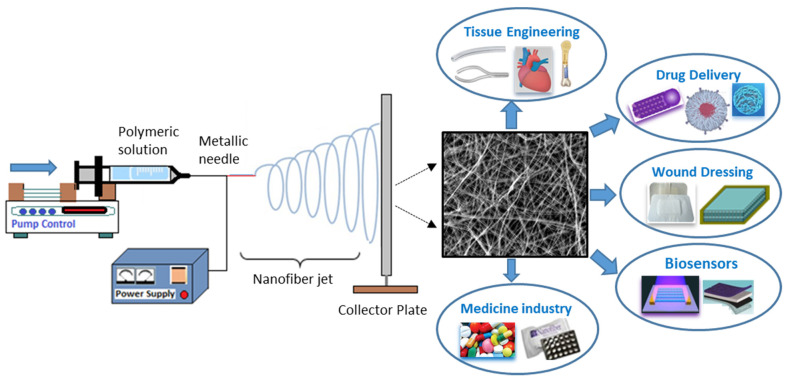
Scheme illustrating electrospun nanofibers process and their related biomedical applications.

Recently, nanofibrous materials via electrospinning technology were widely reported and shown to be effective in various biomedical applications, including tissue engineering, wound dressing, drug delivery, regenerative medicine, disease modeling, and sensing/biosensing [209,210,211,212,213,214,215,216,217,218,219,220] (Figure 7). These sustainable electrospun composite materials were efficient due to their ease of operation, nanoscale diameter, wide specific surface, high porosity, cost-effectiveness, and the large adaptability for engineering eco-friendly bioactive nanomaterials [221,222,223,224,225]. Electrospenning technology has been widely investigated in the field of drug delivery system thanks to its versatility in terms of producing nanofibers which combine several types of products in their initial composition. These nanofibers could be in different morphologies and structures, such as core-sheath [226] and janus [227]; this allows one to control the release rate of the incorporated biological ingredients. Indeed, a wide range of bioactive agents, such as anticancer drugs [228], antibiotics [229], RNA [230], and therapeutic proteins [231], have been successfully immobilized into electrospun polymeric nanomaterials of multiple designs that provide various drug release profiles, including sequential release [232], zero-order release [233], biphasic release [234], spatiotemporal release [235], and stimuli-triggered release [236]. Nowadays, nanofiber delivery systems based on stimuli-sensitive polymers have gained considerable attention in the field of drug delivery systems. These systems were designed to trigger the release of drugs spatiotemporally through chemical or physical stimuli. Several types of stimuli have been explored, including endogenous stimuli such as redox gradient, change in pH, and sensitivity to enzymes, in addition to exogenous stimuli such as magnetic or electric fields, temperature, and light [236,237,238]. Singh et al. [239] prepared a poly(N-isopropylacrylamide) (PNIPAM) composite, stimuli-responsive nanofiber, comprising gold nanorods (GNRs), as an on-demand drug delivery system. Following the near-infrared (NIR) irradiation, the GNRs generate heat, which stimulates a thermal response of the PNIPAM, manifested by the shrinking of the nanofibers and thus the controlled release of the active principle. Cell studies have confirmed the performance of this light-sensitive nanomaterial in revealing its biocompatibility and the efficiency of its swelling and deswelling ability, as well as a controlled release with an efficient on–off behavior. Furthermore, the system has shown its potential in the successful combination of chemotherapy with several drugs to improve the effectiveness of complex cancer treatments. Mamidi et al. [240] designed a pH-responsive composite nanomaterial based on PCL/mercaptophenyl methacrylate grafted carbon nano-onions (f-CNOs), delivering doxorubicin (DOX). Under selected physiological conditions, interactions between DOX and the f-CNOs resulted in the prolonged and controlled release of DOX. Moreover, the nanocomposite exhibits a good cytoxicity and biocompatibility when in contact with human fibroblast cells.

Simultaneously, tissue engineering and regenerative medicine involve concepts bringing together different fields, including chemistry, biology, and materials science engineering. The development of technical and functional materials aimed at replacing or repairing diseased or damaged organs is a multiplex process that requires designed scaffold materials reminiscent of the cellular microenvironment with efficient cell metabolism, vitality, adhesion, and tissue regeneration. Over the past two decades, electrospinning has shown promise in the development of various effective fibrous scaffolds applied in different fields of regenerative medicine, including in dressings, as well as in cartilage, bone, neural, and cardiovascular tissue regeneration.

Indeed, for bone regeneration, many studies have focused on poly-caprolactone (PCL)/hydroxyapatite (HAp), based on nanofibers, and additives which have been studied for scaffold enhancement [241,242,243]. Liu et al. [244] developed a new hybrid bilayer scaffold based on electrospun PCL/gelatine (Gel) nano fibrous material, combined with a 3D printed PCL/Gel/nano-hydroxyapatite (n-HA) scaffold. Promising cell proliferation and adhesion results using L929 fibroblasts were obtained with the heparin-conjugated PCL/Gel nanofibrous material. The hybrid bilayer scaffolding revealed an excellent rate of new bone regeneration. Hashemi et al. [245] designed a 3D polylactic acid/polycaprolactone/gelatin electrospun scaffold with high porosity (80%), and functionalized with ascorbic acid as a bone healing additive. Using a rat calvaria defect model, in vitro and in vivo evaluations of cell proliferation and bone healing were promising, and afford a valuable potential in osteogenesis and cell culture growth. Electrospinning technology was also the most effective strategy for producing nanofiber scaffolds for cartilage regeneration [246,247,248]. A wide range of bioactive molecules, including corticosteroids, drugs, and growth factors have been immobilized in electrospun nanofiber materials for the control of the inflammatory response and the regeneration of new cartilage tissue. Shen et al. [249] prepared an electrospun porous PLA/gelatin nanofibrous scaffold functionalized with chondroitin sulfate (CS), known to be used in the clinical treatment of cartilage disorders. The designed nanocomposite scaffold has demonstrated good mechanical and biocompatible properties, excellent cell proliferation, and crucial inflammatory inhibition. An in vivo study of rabbit cartilage defects revealed clear cartilage repair and a highly anti-inflammatory effect via the reduction of iNOS and PGES, enzymes producing NO and PGE2, respectively, by immunohistology. Chen et al. [250] fabricated a 2D poly (L-lactide-co-ε-caprolactone)/silk fibroin (PLCL/SF) (2DS) electrospun scaffold crosslinked with hyaluronic acid (HA) to further mimic the microarchitecture of native articular cartilage. The in vitro and in vivo evaluation of the 3D HAS biomimetic scaffold was promising and confirmed its ability as a potent candidate for cartilage tissue regeneration applications. To date, electrospinning has gained prominence in the production of innovative scaffolds for the regeneration of myocardial, valve, and vascular tissue. Eom et al. [251] has developed a multi-layered anisotropic scaffold with a 3D anisotropy comparable to that of native heart tissue, based on a polycaprolactone (PCL) nanofiber mat. In vitro cell culture of cardiomyocytes showed the spontaneous contraction of the prepared scaffold mat and demonstrated cell alignment and subsequent uniaxial contraction with aligned nanofibers. The stacked triple layers also exhibited multiaxial contraction, which potentially simulates the compressive force of the heart tissue. Numerous studies have reported the production of various electrospun nanofibrous scaffolds to be applied as skin substitutes and wound dressings [252,253,254]. Choi et al. [255] designed an electrospun polycaprolactone (PCL)/keratin scaffold as a multi-layered skin substitute, mimicking the real multi-layered skin anatomy. The PCL/keratin scaffold revealed good cell adhesion and proliferation on contact with a co-culture of keratinocytes and fibroblasts. In addition, in vivo assays showed the rapid regeneration of new skin without scar formation. Hivechi et al. [256] prepared an exopolysaccharide/PCL/gelatin electrospun nanofibrous material as a skin substitute. The nanocomposite scaffold showed good biocompatibility and cell viability behaviors. The in vivo implantation of the electrospun nanofiber on the full-thickness wound on rat models exhibited rapid healing efficiency.

## 5. Industrial Water Treatment Applications

**Figure 8:**
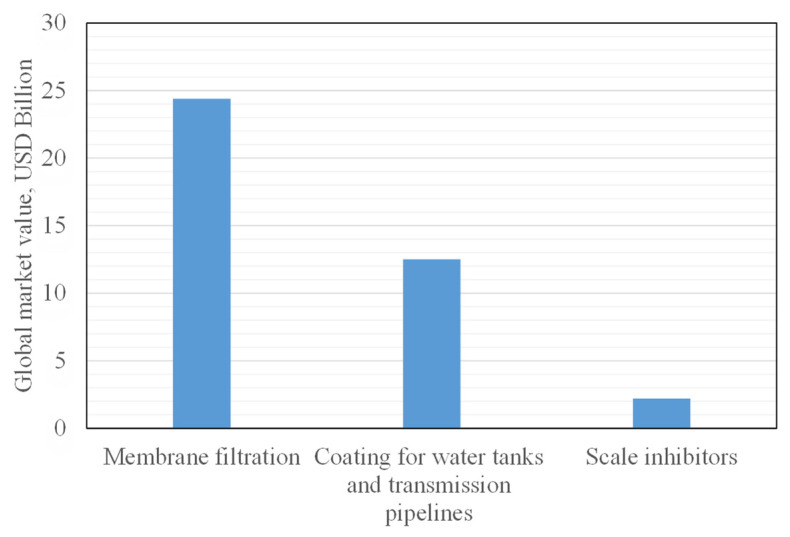
Global market value of some industrial polymeric products.

Polymers play an important role in the water sector. They could be used as chemical additives (as soluble polymers) in water treatment and desalination plants to reduce scale formations and increase water productivity, as coating material to protect water tanks and transmission lines from corrosion, or as materials for membrane manufacturing for water treatment and desalination applications. Another recent investigation of polymers and functional polymers concerns their use in the adsorption of different organic and metallic pollutants from industrial wastewater. Below is a brief overview of the global market value and some examples of polymeric materials that can be used in the water sector (Figure 8).

### 5.1. Chemical Additives

Chemical additives are used in many water applications, such as water treatment, water desalination, and in oil field activities [257]. The global market of scale inhibitors was worth USD 2.26 billion in 2014 and was projected to increase by 4.73% between 2015 and 2020 [258]. There are increasing demands on the use of soluble polymeric additives that are effective, safe to use, and environmentally friendly. Many soluble polymer additives are available in the market, some of which are used in the water treatment and desalination process as scale coagulant aids and scale inhibitors [259,260,261]. The most effective and famous additives are poly(acrylic acid), poly(methacrylic acid), and poly(Maleic acid) which are widely used as scale inhibitors in thermal plants [262,263,264] for reducing scale formation and increasing process performance. Another kind of polymeric additives for water desalination using membrane processes is polyamino polyether methylenephosphonate (PAPEMP), which is an ideal additive as it controls calcium carbonate and calcium sulfate scale formation and deposition [265,266]. Addition of this chemical could reduce or eliminate acid feed, therefore reducing hazardous risk and increasing water production. Recently, a zero generation (0G) polyamidoamine (0G means a dendrimer with only the central core), as an amine-terminated dendrimer (PAMAM) with a reactive core of 1,3-diaminopropane, has been synthesized for the inhibition of silica scale contaminant. This polymeric membrane has shown excellent scale-inhibiting properties and has shown its effectiveness as a novel water treatment membrane [267].

### 5.2. Polymeric Membranes

Polymeric membrane technologies that have been explored in the treatment and desalination of water are continuously developed and widely studied, with the aim of meeting the global challenges of water security and supply. The relevant polymer-based membranes have today become one of the most interesting materials, exploited in many fields such as water treatment, water desalination, and food processing [268,269,270]. The global membrane filtration market size is expected to reach USD 24.4 billion by 2026 [271]. There are many polymeric materials that can be used for membrane filtration which depends on the application area. They can be used as microfiltration (MF) or ultrafiltration (UF) devices for the removal of suspended solids and microorganisms, or large chemical molecules and colloidal particles. Those membranes are mainly made by polypropylene (PP), poly(vinylidene fluoride) (PVDF) and polytetrafluoroethylene (PTFE), due to their durability and availability, as well as their excellent thermal and mechanical stability.

Other useful applications are nanofiltration (NF) and reverse osmosis (RO) membranes for the removal of dissolved salts from water. Polysulphone (PS) and poly(ethersulphone) (PES) and polyacrylonitrile (PAN) are some examples of the materials that are used as a membranes or membrane substrate for NF or RO applications [272,273,274]. Polyamide or cellulose acetate are examples of polymeric materials that are used for fabricating composite membrane for reverse osmosis or nanofiltration [275,276,277].

### 5.3. Polymers in the Treatment of Industrial Wastewaters

Water pollution by organic matter is a global problem, whose aspects and scope are obviously different according to the level of development of the countries involved. It is important today to work and make every effort to reduce the concentrations of pollutants from industrial wastewater. Pollution by toxic organic waste is more insidious than a direct pollution (odor, cloudiness) because it is less remarkable. The health of living organisms is slowly deteriorating, their lives are shortened, their descendants may be affected by malformations, their probability of being reached by cancer will increase, and the aquatic fauna is not the only one concerned. We ingest these same toxic pollutants without having them in our proximity, through the food chain, by consuming the flesh of these living organisms, vegetables, fruits, vegetables, etc.

Dyes are among the most dangerous organic pollutants, and they are often found in the environment as a result of their wide industrial use. These industrial pollutants are common contaminants in wastewater. The textile, paper-making, cosmetic, pharmaceuticals, food coloring, and pulp industries are reported to be the source of large amounts of pollutant dye discharged into wastewater. These colored wastewater pollutants are toxic and even carcinogenic, posing a serious danger to living aquatic organisms [278,279]. This interesting topic requires the development of various technologies to treat colored waters. Biological treatment and coagulation/flocculation processes are viewed as ineffective to treat soluble dyes [280,281,282]. Adsorption using polymeric materials has appeared to be more effective, as it is simple and economic and it is especially used to remove pollutants, which are not easily biodegradable. Thus, a specific attention is devoted to explore new polymeric adsorbents, which could be cheaper, more proficient, and easily regenerated [283,284,285].

In this sense, several synthetic polymeric adsorbents have been used for the removal of organic dyes from contaminated matters [286,287,288]. Among these synthetic polymers investigated as adsorbents of organic dyestuffs, we can mention the use of PVA, and various composite materials based on PVA [289,290,291,292,293]. Conductive polymers have also been investigated to be effective adsorbents. Indeed, the polyaniline and polypyrrole polymers, as well as their related materials and composites, have revealed in the literature excellent adsorption capacities of various dyes and organic pollutants. [294,295,296]. Synthesized polymers based on cyclodextrins macromolecules have been studied as adsorbents for different basic dyes in aqueous solution. These adsorbents exhibited high sorption capacities [297,298,299]. Magnetic nanoparticles which were modified by different polymers (3-aminopropyltriethoxysilane) and copolymers of acrylic acid, or crotonic acid, have shown promising performances for removing various aqueous pollutants [300,301]. Silver-based coordination polymers were developed and demonstrated good adsorptive performances toward a series of organic dyes with sulfonic groups [302].

In recent years, natural polymeric bio-sorbents are gaining more interest over synthetic classical adsorbents, due to their tunable physicochemical properties, structural diversity, reusability, and environmental benefits [303,304,305]. Indeed, several low-cost polymeric adsorbents have been prepared through the valorization and the functionalization of wastes from different sources, such as plants, fish shells, marine algae (green, brown and red species), vegetables, etc. [306,307,308,309]. These materials are sources of cellulose, chitosan, sodium alginate, carrageenan, lignin, etc. These natural polymers have been exploited either directly after extraction or by mixing them in polymeric composites for more compactness and especially efficiency [310,311,312,313,314,315,316,317]. The adsorption results of various organic pollutants have been very promising in terms of their absorption capacity, simplicity of operation designs, cost-effective aspects, and reuse. [318,319]. Very recently, a new procedure for exploring natural polymers as effective adsorbent materials has been investigated. Thus, certain biodegradable textile polymeric materials, such as cellulose, have been functionalized by different extracted natural polymers (chitosan, carrageenan, alginate, etc.). This is a simple and economical method offering permanent and stable natural adsorbents with high adsorption capacities [320,321].

Hereafter, we present an overview of the various natural adsorbent materials studied in the adsorption of organic waste pollutants (Table 1).

**Figure 9:**
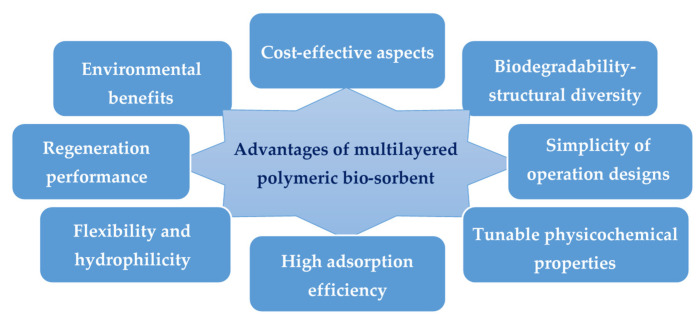
Benefits of natural multilayered polymeric bio-sorbents for the adsorption of organic pollutants from industrial wastewater.

**Figure 10:**
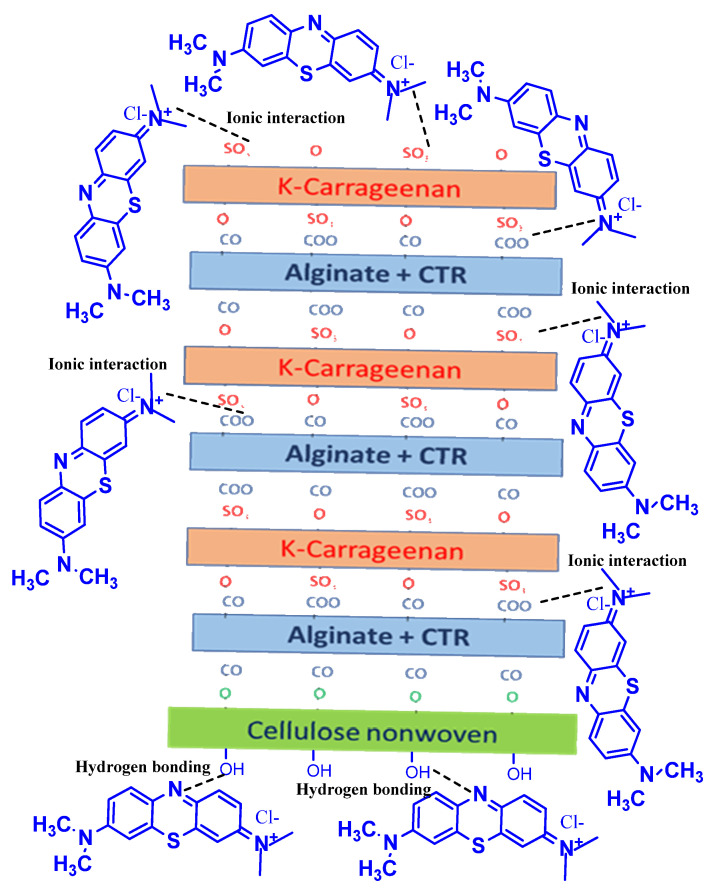
Illustration of different interactions between methylene blue molecule and the surface of multilayered polymeric material (Alginate and Carrageenan) crosslinked to non-woven cellulosic bio-sorbent. Reprinted with permission from MDPI [329], Copyright 2021.

Furthermore, some novel adsorbent designs based on polyelectrolyte multilayered (PEM) bio-polymeric materials as potent bio-sorbents were studied [329,388]. These adsorbents were prepared by an alternation of layers of two polyelectrolyte biopolymers via a layer-by-layer grafting method. Different layers were grafted to a natural cellulosic non-woven material and providing thereby many practical advantages (Figure 9). Various natural polymers were investigated and combined, such as chitosan, alginate, carrageenan, etc. Polyanionic polymers were effective in adsorbing positively charged molecules (Figure 10) and grafted polycationic polymers demonstrated an excellent performance in removing negatively charged molecules and dye wastes. Overall, the covalent immobilization of the biopolymer on the surface of the material has been beneficial in ensuring the successful recycling and reuse of bio-sorbents without significant loss in adsorption performance. The adsorption capacity results were therefore very interesting, in addition to their low cost and easy reuse.

Finally, due to the specific selectivity of polymeric materials, which are functionalized via a wide variety of polymers having various active groups, such as amines, hydroxyl, carboxylic, phosphonic, and sulfonic, towards the target pollutant, particular attention will be given to these.

### 5.4. Other Applications in Water Sector

Protecting water tanks and transmission pipelines with a properly coated material, to avoid or minimize corrosion, is essential for extending the lifetime of storage tanks and pipes, as well as for maintaining water quality. Nowadays, there are many coating materials, such as acrylic, polyurethane, epoxy, etc. that are used for many industrial and marine works. The market size is estimated at USD 8.7 billion in 2018 and is projected to reach USD 12.5 billion by 2023 [394]. Polyurea is also perfect for water tank lining, due to its elasticity, thermal stability, and ease of use. Polyurea can be effective in protecting steel, aluminium, and fiberglass in a variety of water activities and commercial marine applications [395,396]. These chemicals, or other alternative chemicals with similar properties and usage, could be formulated and manufactured to cover industrial demands and to increase the business in the local contents of these materials. Functional polymers and their composites were also applied for water treatment and desalination. Different polymeric composites were thus prepared and studied, such as polymer-carbon composites [397], polymer graphene composites [398], polymer-based activated carbon composites [399], and polymer anionic/cationic clay composites [400].

The development of polymeric materials is becoming the most promising future alternative to meet environmental water standards and to grant the water needs of growing populations.

## 6. Conclusions

As shown in this review, synthetic and natural polymers exhibit excellent effective performance in many areas of application. Recent continuous development in various polymer functionalization technologies and nanotechnologies have endowed polymeric materials with more interesting properties and tracing functionalities, promising to overcome the various problems and shortcomings downgrading the impact and the efficiency of polymers in their applications. Therefore, the simple combination of various native polymers cannot fulfill all the required properties, and thus, surface functionalization represents an efficient alternative and a crucial strategy that grants new a performance with added value. In this perspective, the functionalization methods emerge as a basic line for the obvious improvement of the functionalities of these applied materials. Despite the significant progress in recent years and the high level of success of polymer functionalization in different fields, several challenges still remain, and there is room yet for various improvements. First of all, a comprehensive and precise knowledge of the biological mechanisms of cell–polymer interactions could guarantee better design improvement. Indeed, many pathways related to cell uptake, cell adhesion kinetics, cytotoxicity, etc., are still under study and a common theory should be established. Concerning energy applications, as discussed, various studies have indeed confirmed the great potential of functional polymer materials for the production and storage of sustainable energy. However, their use for such applications has encountered greater challenges in their commercial adoption, due to the lack of strategies that converge manufacturing speed and precision to produce cost-effective, efficient, and selective devices. In addition, further studies are still needed to assess the long term stability of such polymer systems.

Furthermore, the passage from the laboratory scale for the various polymer applications in industrial production is not evident, and still has many deficiencies and gaps. In addition, there is typically the problem of a large delay, of great requirements, and complicated laws for a finished product to be industrialized and commercially available.

In summary, by solving all these problems and overcoming the various obstacles, we can affirm that natural and synthetic functional polymers and their composites represent a milestone for diverse energy, environmental, and biomedical applications and they guarantee an optimistic and promising future.

## Figures and Tables

**Table 1:** A synthesis of the various recent natural polymeric bio-sorbents and their composites studied for the adsorption of anionic and cationic dyes from industrial wastewater.

Adsorbent	Adsorbate	q_t_ (mg·g^−1^)	Adsorption Efficiency (%)	References
Luffa Cylindrica fibers	Malachite green/MB	63/52	96.72/94.40	[322]
Bean peel	Cibaron Blue	5.72	95.31	[323]
Eichhornia crassipes roots	4B red reactive	43.28	95	[324]
Dead Typha angustifolia (L.) leaves	MB	106.75	89.83	[325]
Almond shell	MB	84.9		[326]
Salix babylonica leaves	MB	60.97		[327]
Prickly (peel) bark of cactus fruit	MB	222		[328]
k-carrageenan/alginate/cellulose PEM	MB	522.4	98.6	[329]
Coconut mesocarp	Remazol golden yellow	6.8	94.9	[330]
	Reactive gray BF-2R	4.8	100	
	Reactive turquoise Q-G125	4.1	96.6	
Banana peels	Remazol golden yellow	2.5	70.2	[330]
	Reactive gray BF-2R	1.2	75.4	
	Reactive turquoise Q-G125	1.6	100	
Typha australis Leaves	Malachite green	85.21	-	[331]
white pine (Pinus durangensis) sawdust	MB	87	86	[332]
pineapple (Ananas comosus) leaf	Remazol Brilliant Blue R	9.66	96.20	[333]
Lime (Citrus aurantifolia) peel	Remazol Brilliant Blue R	9.58	95.89	[333]
Coconut bunch	Remazol Brilliant Blue R	9.48	94.76	[333]
Coconut fiber	Remazol Brilliant Blue R	9.55	95.48	[333]
Chili seeds	Remazol Brilliant Blue R	9.40	93.97	[333]
Guava leaves	Remazol Brilliant Blue R	9.41	94.09	[333]
Coconut residue	Remazol Brilliant Blue R	9.38	93.85	[333]
Jackfruit peels	Remazol Brilliant Blue R	9.23	92.26	[333]
Orange peel	Remazol Brilliant Blue R	-	11.4	[334,335]
Spent tea leaves	Remazol Brilliant Blue R	9.7	-	[334]
Salvinia natans	Remazol Brilliant Blue R	61.9	-	[336]
Durian peel	Remazol Brilliant Blue R	-	14.9	[336]
watermelon rind	MB	200	99	[337]
	Brilliant green	188.6	98	[338]
	Remazol Brilliant Blue R	333.33	92–97	[339,340]
	Congo red CR	17	100	[341]
	Orange G	27	85	[342]
Cyanthilium cinereum L. H. Rob weeds	MB	76.336	95	[343]
Paspalum maritimum (PMT)	MB	56.179	97	[343]
Carica papaya wood	MB	32.25		[344]
pupunha palm	MB	78.989		[345]
Potato shell	MB	48.7		[346]
Scenedesmus	MB	61.69		[346]
Maize silk powder	MB	132.1		[347]
Lignin sulfonate polymer	Malachite green	27.4	60	[348]
Lignin sulfonate-*g*-poly(acrylic acid-*r*-acrylamide)	Malachite green	97	97	[349]
Microalgae *Scenedesmus*	MB	87.69	-	[350]
activated carbon prepared from Date Press Cake	MB	613.8	83.3	[351]
Karanj fruit hulls	MB	239.4	94.4	[352]
Rattan (*Lacosperma secundiflorum*)	MB	359	96	[353]
Fox nutshell	MB	968.7	99.96	[354]
chitosan-epichlorohydrin/zeolite composite	MB	44.2	90	[355]
	Reactive red 120	45.25	88	
chitosan/carboxymethyl cellulose capsules	MB	64.6	4.4	[356]
	Methyl orange	334.8	37.5	
	Acid blue-113	526.8	59	
Polyacrylamide-chitosan magnetic nanoparticles	MB	1044.06	76.1	[357]
chitosan-epichlorohydrin/TiO_2_ nanocomposite	Reactive red 120	46.3	99.3	[358]
polypyrrole-chitosan composites	Acid red 18	285.71	98.93	[359]
Chitosan-Activated Charcoal Composite	Thionine cationic dye	60.9	92.9	[360]
chitosan-glyoxal/TiO_2_ nanocomposite	Methyl orange	374.8	75.9	[361]
chitosan/polyamide nanofibers	Ponceau 4R	482.2	-	[362]
	Reactive Black 5	352.5	-	
Chitosan/alginate composite sponge	Acid red B14	1486.9	-	[363]
fibrous chitosan/alginate composite foam	MB	1488.1	-	[364]
	Acid Black-172	817	-	
Sodium alginate nanofiber membranes	MB	2230	-	[365]
Sodium alginate/gelatin/graphene oxide composite aerogel	MB	322.6	-	[366]
	Congo red	196.8	-	
Lignin/cellulose nanocrystals/alginate beads	MB	1181	-	[367]
Cladodes of *Tacinga palmadora*	Crystal violet	228.74	-	[368]
Palm cactus	Crystal violet	220	-	[369]
*O. ficus indica* cladodes	Acid orange	198.9	-	[370]
Cactus pear seed cake	MB	260	56.48	[371]
	Methyl orange	336.12	100	
Fruit peels (*O. ficus indica*)	Indigo carmine	294	76–99	[372]
	Solophenyl blue	909	76–99	
	MB	416	76–99	
	Crystal violet	312	76–99	
Carboxymethyl chitosan-modified magnetic-cored dendrimers	MB	20.85	-	[373]
	Methyl orange	96.31	-	
Gelatin-based magnetic beads	MB	465	-	[374]
	Direct Red	380	-	
Glutaraldehyde cross-linked chitosan-coated Fe_3_O_4_ nanoparticles	Methyl orange	758	-	[375]
Magnetic hydrogel beads with gum tragacanth	Congo Red	94	-	[376]
Fe_3_O_4_–κ-carrageenan cross-linked with chitosan	MB	123		[377]
Fe_3_O_4_@SiO_2_–κ-carrageenan	MB	530	-	[378]
Potamogeton crispus	RR198	44.2	-	[379]
O-carboxymethylchitosan-N-lauryl/γ-Fe_2_O_3_ magnetic nanoparticles	RR198	216	-	[380]
Pistachio hull wastes	RR198	253.67	95.13	[381]
Al_2_O_3_/MWCNTs Carbon nanotube	RR198	424	91.54	[382]
Polyaniline/Fe_3_O_4_	RR198	45.45	92.1	[383]
Eggshell biocomposite beads	RR198	46.9	92	[384]
Activated Carbon (Walnut Shells)	RR198	79.15	87.17	[385]
Pistachio nut shell	RR198	108.15	88	[386]
Chitosan	RR198	310.4	95.11	[387]
Chitosan/cellulose PEM	RR198	819	99.77	[388]
Cellulose/chitosan aerogels	Congo Red	381.7	95	[389]
Chitosan/Zeolite composite	MB	19.23	84.85	[390]
Chitosan/PVA composite	Methyl orange Eosin Yellow Dye	9.34 52.91	92.42 86.70	[391] [392]
Chitosan/ZnO	Malachite Green Dye	-	98.50	[393]

## Data Availability

The data presented in this study are available on request from the corresponding authors.
